# Directed Self-Assembly of Block Copolymers for the Fabrication of Functional Devices

**DOI:** 10.3390/polym12102432

**Published:** 2020-10-21

**Authors:** Christian Pinto-Gómez, Francesc Pérez-Murano, Joan Bausells, Luis Guillermo Villanueva, Marta Fernández-Regúlez

**Affiliations:** 1 Instituto de Microelectrónica de Barcelona (IMB-CNM, CSIC), Campus UAB, 08193 Bellaterra, Spain; christian.pinto@imb-cnm.csic.es (C.P.-G.); francesc.perez@csic.es (F.P.-M.); joan.bausells@imb-cnm.csic.es (J.B.); 2 Advanced NEMS Laboratory, École Polytechnique Fédérale de Lausanne (EPFL), CH-1015 Lausanne, Switzerland; guillermo.villanueva@epfl.ch; 3 Universitat Autònoma de Barcelona, 08193 Bellaterra, Spain

**Keywords:** block copolymers, directed self-assembly, graphoepitaxy, nanolithography, nanomechanical devices, nanowires

## Abstract

Directed self-assembly of block copolymers is a bottom-up approach to nanofabrication that has attracted high interest in recent years due to its inherent simplicity, high throughput, low cost and potential for sub-10 nm resolution. In this paper, we review the main principles of directed self-assembly of block copolymers and give a brief overview of some of the most extended applications. We present a novel fabrication route based on the introduction of directed self-assembly of block copolymers as a patterning option for the fabrication of nanoelectromechanical systems. As a proof of concept, we demonstrate the fabrication of suspended silicon membranes clamped by dense arrays of single-crystal silicon nanowires of sub-10 nm diameter. Resulting devices can be further developed for building up high-sensitive mass sensors based on nanomechanical resonators.

## 1. Introduction

In standard micro/nanofabrication, structures and devices are created by depositing a variety of thin films, followed by pattern definition by lithography and etching to remove undesired regions of material. Decades of development in each of these three main steps have generated technological advances that have enabled a relentless miniaturization of microelectronics devices, keeping Moore’s Law and the race towards shrinkage alive [1,2].

The driving force of this steady reduction in feature size has been the evolution of lithography, as improvements in optical lithography have provided sufficient resolution to reach the 10 nm semiconductor node by means of deep ultraviolet (DUV) immersion lithography [3,4,5,6]. However, for further scaling, light diffraction limitations have led to the exploration of new lithographic solutions [7], which include extreme ultraviolet lithography (EUV) [8], nanoimprint lithography [9], multi-beam electron-beam lithography [10] and directed self-assembly (DSA) of block copolymers (BCPs) [11].

As of today, EUV has taken over the main spot for advanced lithography at the industry level and has already been introduced in production lines of nanoelectronic devices. As EUV and its combination with multiple patterning presents exponentially increased costs due to its extreme complexity, research in other less conventional lithographic techniques still remains of great interest.

It is in this scenario where DSA is considered as an attractive alternative for the fabrication of nanoscale structures, thanks to its high resolution, low cost, ease of integration and scalability [12,13,14]. Being an affordable high-resolution method with the possibility of scaling up, it has drawn a lot of attention in industrial semiconductor processing since the late 1990s [15,16,17], long before the commercial availability of EUV [18,19,20]. However, the present main limitation for its further incorporation into high-volume manufacturing is its capability to meet industry defect density standards [21,22].

DSA is based on the ability of BCPs to phase separate spontaneously. BCPs are macromolecules consisting of covalently bonded homogeneous blocks (or chains) of chemically different monomers. Due to the dis-affinity and repulsion forces between these blocks, and in order to present minimal free energy, BCPs segregate into microdomains after a thermally-driven phase separation process. They generate self-assembled patterns within the nano/microscale [23], which facilitates their use in high-resolution nanopatterning [24]. After self-assembly, one of the blocks is removed and the remaining polymer is used as mask to pattern the substrate underneath [25]. Furthermore, DSA has been incorporated into conventional 300-mm pilot lines with existing tracks for logic applications [26,27,28], and also in a variety of other applications, such as non-volatile memory, sensors, photovoltaics, solar cells, graphene patterning or liquid separation membranes [29,30,31].

## 2. Principles of the DSA of Block Copolymers

### 2.1. Phase Segregation in Diblock Copolymers

The simplest BCPs are linear A-b-B diblock copolymers, where A and B are two different polymeric blocks, joined together by a covalent bond. Three parameters determine the period, morphology and phase behavior: the total number of monomers forming the BCP (degree of chain polymerization, N), the relative volume fraction of each block (f) and the Flory-Huggins interaction parameter (χ) [23,32]. This parameter is inversely proportional to temperature [33], depends on BCP chemistry and gives an idea of how strong the repulsive force between the blocks is.

The microphase segregation strength of BCPs is usually expressed by the product χN [34] and, in order to observe an ordered state, it needs to be above the critical lower limit of χN = 10.5 [35]. For values below that, clear phase segregation does not occur. Therefore, to obtain structures with small period from BCPs with low N, high immiscibility between blocks is desired [36]. Finally, for a certain χN, polymer self-assembly will occur in different shapes depending on f. For instance, the microphase separation of an A-b-B diblock copolymer can take place in geometries like closed-packed spheres, hexagonally packed cylinders, body-centered spheres, gyroid or lamellae, depending on the relative volume fraction of blocks A and B [37].

An example of a diblock copolymer is polystyrene-block-poly(methyl methacrylate), PS-b-PMMA, formed by a chain of PS and a chain of PMMA covalently bonded. This BCP is amongst the most studied for nanolithography applications, as both blocks are polymers with well-known etch properties, easy to handle, present reasonable temperature ranges for annealing and similar affinity to air [38,39,40]. Extensive characterization of self-assembled PS-b-PMMA thin films has been carried out regarding annealing conditions, kinetics, defectivity, line-edge roughness and nanomechanical properties of the blocks [22,41,42,43,44,45,46,47,48,49].

### 2.2. Self-Assembly on Thin Films

When a diluted solution of BCP is spin-coated on a substrate in the form of a thin film and self-assembly is induced, microphase separation takes place in short-range order, generating condensed arrays of random periodic structures. In this situation, phase behavior is strongly influenced by surface energetics [50], and domain orientation will arise determined by the relative strength of the surface affinity to each block of the BCP. If the substrate shows higher affinity to one of the blocks, it will attract the domains with lower interaction energy stronger than the others, in an effort to keep free energy minimum at the polymer-substrate interface. This can cause the BCP to self-assemble in an undesired morphology that might be useless for nanolithography applications, for example.

**Figure 1:**
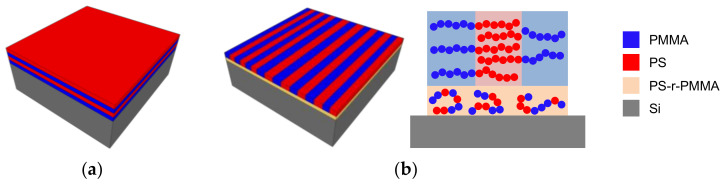
Schematic of the self-assembly of lamellar PS-b-PMMA. (**a**) If the substrate shows preferential interaction with PS or PMMA, self-assembly in horizontal lamellae guarantees minimal free energy at the interface; (**b**) a random copolymer brush (PS-r-PMMA) can be used to impel neutral affinity to the substrate, coercing the generation of vertical PS and PMMA lamellae.

To avoid this, the most straightforward way to control pattern orientation in the self-assembly of a BCP thin film is to balance the surface free energy between domains by spin-coating a random copolymer composed of the same monomer units as the block copolymer (PS-r-PMMA for PS-b-PMMA) in between substrate and BCP [38,51] (Figure 1). This thin film is known as neutral layer or brush, and by tweaking its composition the interface energy can be modified to favor the affinity of the substrate to one block or the other. Then, by applying a certain temperature higher than the glass transition temperature of both blocks or by placing the sample in a solvent atmosphere [52], homogeneous phase separation can be achieved.

### 2.3. Enabling Lithography: DSA of BCPs

In order to be useful for lithographic purposes, BCPs must be someway guided into the desired long-range order and morphology. To do so accurately, templates known as guiding patterns (GPs) are used to direct the self-assembly, whilst BCP properties (molecular weight and composition) and thermodynamics control the feature size, shape and uniformity of the resulting features. As the density of GPs is generally lower than the pitch of the self-assembled microdomains, BCPs are a valuable pattern multiplication method that is able to provide resolution enhancement to pre-patterned templates [53,54].

**Figure 2:**
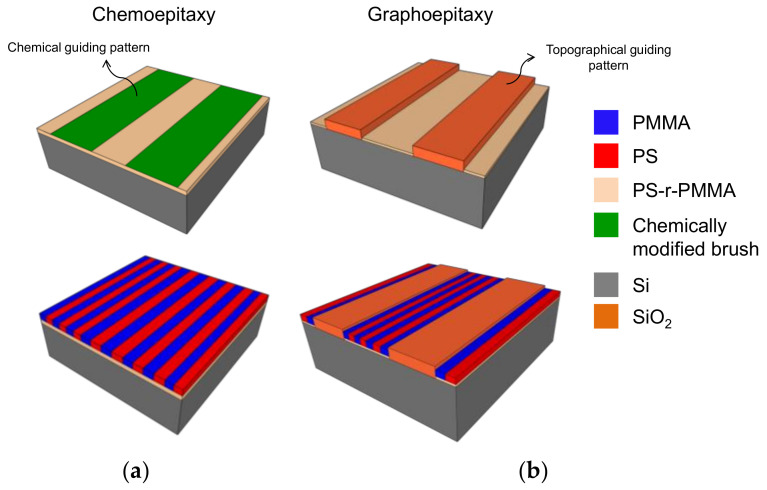
Schematic of DSA by chemo- and graphoepitaxy. (**a**) In chemoepitaxy, areas of the substrate are chemically activated to show stronger affinity to one of the blocks, directing the self-assembly; (**b**) in graphoepitaxy, the substrate is topographically structured to direct the self-assembly.

GPs are normally fabricated by top-down techniques, following two different approaches: chemoepitaxy and graphoepitaxy (Figure 2) [11,55,56]. Chemoepitaxy involves the creation of dense chemical patterns on a neutral substrate to generate preferential wetting sites for one of the blocks [57]. Multiple processes and techniques have been successfully used to selectively tune the surface free energy of a neutral surface, including photolithography [58], electron-beam lithography (EBL) and oxygen plasma functionalization [59,60,61,62,63], direct EBL exposure [64] and scanning-probe lithography [65,66,67].

Graphoepitaxy, on the other hand, is based on the definition of 3D features on the substrate, within DSA takes place [68,69,70,71,72]. These topographical templates can be physically tailored, and bottom and walls along the trenches chemically modified to impose different affinity to each of the polymer blocks, enforcing their orientation along the topography [73,74].

### 2.4. Present Directions

The low value of χ in PS-b-PMMA limits minimum attainable resolution (to about 22 nm) [20,75]. Solutions to overcome this limitation come from chain modification of well-known BCPs [76,77], the use of additives [78] or the pursuit of novel molecular architectures of high-χ BCPs, which combine polymers that are strongly immiscible, giving access to sharper phase-separation at smaller natural periods [79,80].

A wide variety of high-χ BCPs have been synthetized in the last few years, including organic and inorganic species [36,81,82,83,84,85]. Examples include organic polystyrene-b-poly(2-vinyl pyridine) (PS-b-P2VP) [86,87] or polystyrene-b-poly(propylene carbonate) (PS-b-PPC) [88], and inorganic polystyrene-b-poly(trimethylsilylstyrene) (PS-b-PTMSS) [89] or polystyrene-b-polydimethylsiloxane (PS-b-PDMS) [90], for instance.

The inclusion of inorganic blocks in BCPs is particularly interesting as they increase dis-affinity and provide higher etch contrast during selective block removal and pattern transfer [81,82,83,91]. The implementation and processing of such BCPs is not easy, nonetheless. For example, the lower surface energy of inorganic blocks can make it necessary to increase the number of etching steps for the pattern transfer due to the presence of preferential wetting layers at both air/polymer and substrate/polymer interfaces [92]. High-χ BCPs usually present low tolerance to high temperatures and tend to organize differently on the substrate surface than at the air surface, commonly requiring solvent annealing [93,94,95] or an overcoat of polymer to assure structures are standing up in the final BCP film [84,96,97].

Additionally, recent research on DSA has also been greatly focused on the study, understanding and mitigation of defects [98,99,100,101,102,103,104,105,106,107]. Minimization of defects is crucial for the incorporation of DSA into high-volume manufacturing, either as a primary patterning option or in combination with already established techniques like DUV or EUV.

## 3. Block Copolymers for the Fabrication of Functional Devices

Line-space pitch multiplication and contact via level patterning are the two pivotal applications of DSA in high-volume manufacturing. In line-space applications lamellar BCPs have been used as mask for the definition of arrays of silicon fins that constitute the central body of non-planar fin field-effect transistors (FinFETs). Multiple works have demonstrated the capability of DSA to fabricate silicon fins [108,109,110,111,112,113], with probably the two most well-known being the LiNe and IBM lift-off chemoepitaxial processes. In the LiNe process a cross-linked polymer mat is deposited on the substrate and then patterned by photolithography to define the GPs. Afterwards, the interspatial regions where the base polymer was removed after patterning are refilled with a neutral brush layer, followed by self-assembly of the BCP [114,115,116]. On the other side, in the IBM lift-off process sacrificial features are created by photolithography, followed by neutral layer deposition and lift-off, leaving both neutral brush and substrate areas with preference to one of the two blocks, exposed [58,117].

Regarding graphoepitaxial approaches, the most common strategy is to use topographical GPs in the shape of trenches, balancing the surface free energy between BCP domains and bottom of the trench, while un-grafted GP walls show stronger affinity to one of the blocks [39,118,119,120,121]. As consequence, when DSA is performed, domains are aligned perpendicularly to the bottom surface and parallel to the walls. However, this methodology presents several drawbacks. First, defects might occur due to local variation of affinity in areas of the sidewall. Second, high-resolution lithography is required for the fabrication of the GPs, as only few lamellae are normally possible to align parallel to the GP walls with low defectivity. Finally, great control in the deposition of the brush layer is needed to only graft it on the bottom of the trench and not on the sidewalls.

In contact via level patterning cylindrical or lamellar BCPs can be integrated directly into conventional CMOS lithography to generate contact-hole shrinking, contact multiplication or contact uniformity enhancement [122,123]. Firstly, GPs are pre-patterned using optical lithography (or EBL), followed by dry etching for their structuring. Afterwards, the surface of the cavity that serves as GP is tuned to be attractive to PMMA. Then, the BCP is spin-coated filling the GP and self-assembly is carried out by thermal annealing. Finally, PMMA is etched away, and the remaining PS is used as mask for pattern transfer of shrunk uniform holes [124,125].

The fabrication of silicon vertical structures in the form of nano-sized pillars is another promising target of DSA processes in nanoelectronics. Pillar fabrication has been demonstrated by combination of DSA with tone-inversion [126,127], by sequential infiltration synthesis in BCPs [128,129], and directly by pattern transfer of the BCP template [130]. As we approach the most extreme semiconductor nodes in terms of scaling, alternative architectures and devices such as vertical gate-all-around field-effect transistors (GAA FETs) or single-electron transistors (SETs) are entering into discussion [131], which could be potentially fabricated by DSA.

Furthermore, besides DSA for logic, research efforts in BCP technology have lately centered their attention on other emerging areas that were looking for large area nanostructuring techniques. Many of these fields are low demanding regarding BCP defectivity levels and, in many cases, self-assembly does not require to be directed, but rather take place on a free surface without guidance. Applications include, but are not restricted to, hard-disk drive and magnetic storage devices [126,132,133,134,135], nanophotonics and plasmonics materials [136,137,138], or chemical sensors [139]. Most often, BCPs are still used as templates for patterning, as in the case of graphene structuring [140,141,142,143], the fabrication of nanoporous membranes [144,145,146,147,148] or energy storage, photovoltaics and batteries [149,150,151,152]. In other applications, however, BCPs can present a more active role and can be used as stabilizing agent, for surface functionalization [153,154,155,156] or to aid in nanoparticle self-assembly [157].

## 4. DSA of BCPs for the Fabrication of Nanoelectromechanical Devices

Nanoelectromechanical systems (NEMS) are interesting building blocks for the realization of sensors as the properties conferred by their extremely reduced dimensions, large surface area-to-volume ratios and minimal masses allow obtaining ultrahigh sensitivity [158]. In particular, nanowires and membranes are functional structures to fabricate nanomechanical resonators. Being very small, they are easy to control electrostatically and show large resonant frequencies which, for instance, make them ideal for the fabrication of high-resolution mass sensors [159,160], where the shift in resonant frequency is an indication of a change in mass [161,162,163,164]. A wide spectrum of materials and techniques has been studied for their fabrication, including traditional micro/nanoelectronics top-down techniques and bottom-up nanofabrication approaches using atoms or molecules as aggregated blocks [165]. However little to no results can be found on the exploration of DSA of BCPs for the definition of NEMS.

**Figure 3:**
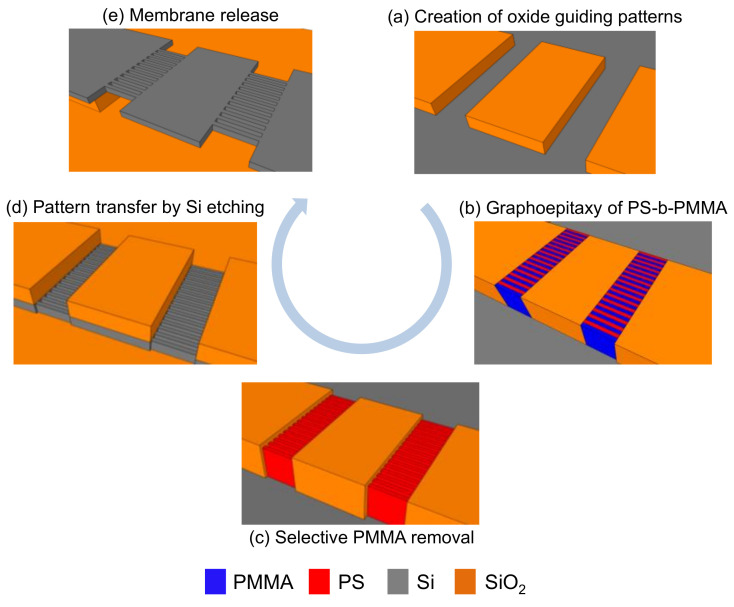
Main steps of the process flow developed for the fabrication of suspended SiNWs and membranes. (**a**) Silicon oxide GPs are created by EBL; (**b**) graphoepitaxy of PS-b-PMMA is performed in such a way that lamellae become perpendicular to walls and bottom of the trenches; (**c**) PMMA is selectively removed by dry etching; (**d**) remaining PS and SiO_2_ are used as mask to define SiNWs and silicon membranes, respectively; (**e**) structures are released from the BOX.

Here we present a DSA process based on the graphoepitaxy of lamellar PS-b-PMMA, which enables the fabrication of ultra-thin silicon membranes suspended by high-density arrays of silicon nanowires (SiNWs) (Figure 3). Each step of the fabrication process is compatible with standard CMOS technology and can be scalable to high-volume manufacturing.

Nanowires obtained through this process display sub-10 nm diameters. Such small dimensions and reduced mass shoot their resonant frequencies up to values where their characterization as resonators themselves would not be trivial. To soften these conditions, devices were devised as a combination of arrays of sub-10 nm SiNWs and larger silicon membranes. The addition of these bigger structures as part of the final design reduces the resonant frequency of the system by increasing the total mass [166,167].

Firstly, SiO_2_ structures are created on top of a silicon-on-insulator (SOI) substrate by EBL, generating walls and trenches that will aid as GPs for graphoepitaxy. In addition, these structures not only serve as GPs for DSA, but also as mask to define the membranes. Then, graphoepitaxy of lamellar PS-b-PMMA is performed after previously grafting a neutral layer all over the GP surfaces. After selectively removing PMMA, PS and SiO_2_ are directly transferred onto the device layer of the SOI substrate by dry etching, creating the SiNWs and the silicon membranes. Finally, devices are released and suspended by attacking the buried oxide (BOX) in hydrofluoric acid.

### 4.1. Creation of Oxide GPs by EBL

As DSA was going to be carried out by graphoepitaxy, topographical GPs were created on top of the substrate to control and lead the BCP to self-assemble in the desired direction and orientation. We decided to define GPs by EBL, mainly due to its flexibility for prototyping. The starting substrates were SOI 2 × 2 cm^2^ chips with an ultra-thin top Si layer of 25 nm (BOX thickness of 152 nm), which defined final device thickness.

First, chips were cleaned by oxygen plasma at 600 W and dehydrated in a short baking step. Later, a single layer of 2% hydrogen silsesquioxane (HSQ) solution in methyl isobutyl ketone (MIBK) (XR-1541, Dow Corning, Midland, MI, USA) was spin-coated at 1500 rpm for 1 min, followed by a bake of 4 min at 80 °C on a hot-plate. When areas with HSQ are exposed with enough dose, it cross-links and transforms into a SiO_x_ material, similar to silicon oxide [168].

GP designs were patterned in a Raith 150^TWO^ tool (Raith GmbH, Dortmund, Germany) at 30 kV, with a 10 μm aperture and dose of 940 μC/cm^2^. After exposure, HSQ was developed in tetramethylammonium hydroxide (TMAH) 25% at 50 °C for 75 s, followed by a strong rinse in deionized water, a dip in isopropanol (IPA) and dried in N_2_.

**Figure 4:**
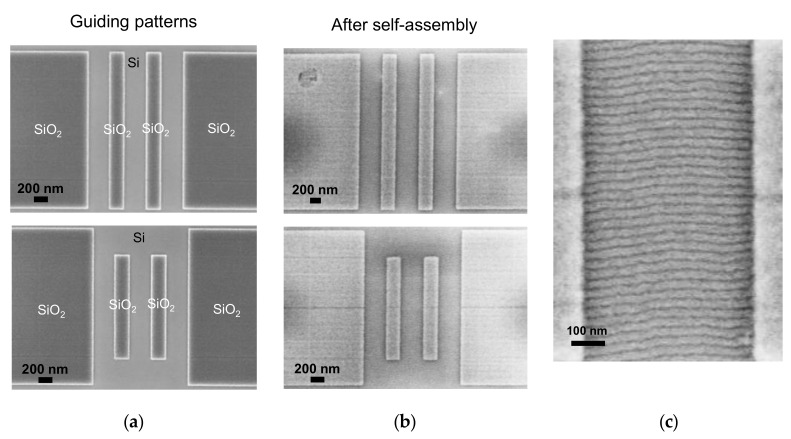
(**a**) Scanning electron microscopy (SEM) top-view micrographs of HSQ GPs after development; (**b**) SEM top-view micrographs after graphoepitaxy within GPs. Parallel PS-b-PMMA lamellae (28 nm pitch) can be observed perpendicular to the walls and bottom of the trenches; (**c**) SEM top-view micrograph of a 500 nm trench after graphoepitaxy of PS-b-PMMA (28 nm pitch).

Square and rectangular oxide-like GPs were fabricated (Figure 4a) maintaining trenches for graphoepitaxy with widths between 350 nm and 550 nm to ensure defect-free perpendicular alignment of PS-b-PMMA. Final GP height after exposure and development under these conditions was ∼30 nm.

### 4.2. Graphoepitaxy of PS-b-PMMA

**Figure 5:**
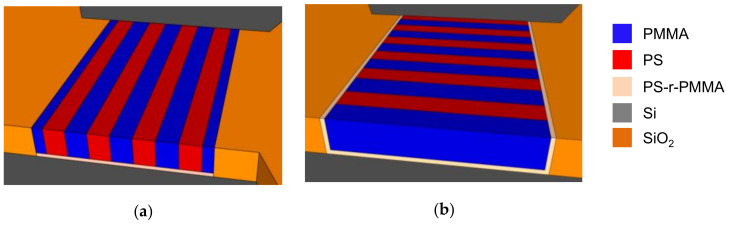
Graphoepitaxy of lamellar PS-b-PMMA within silicon oxide trenches. (**a**) If the bottom of the trench is neutral, but walls are affine to one block, vertical lamellae self-assemble parallel to the walls; (**b**) if walls and bottom are neutral, vertical lamellae self-assemble perpendicular to the three surfaces.

As introduced in Section 3, usually in graphoepitaxy a neutral layer is deposited on the bottom of the trench, in order to balance the free energy between surface and BCPs [169,170] (Figure 5a). In this case, self-assembly occurs in vertical lamellae parallel to the walls and perpendicular to the bottom, in an interesting morphology for the pattern transfer of lines and spaces. Nevertheless, as mentioned, high-resolution lithography to fabricate the trenches and extreme control in the deposition of the neutral layer to graft it only at the bottom are needed.

In the process flow we present, we suggest a much less-demanding methodology in terms of GP resolution and brush deposition (Figure 5b). Here, by spin-coating a thick layer of random copolymer, we completely cover the GPs, grafting the brush on all the surfaces of the trench. As bottom and both walls present neutral affinity, after BCP annealing, PS and PMMA lamellae become perpendicularly aligned to bottom and sidewalls. This approach is much less strict, as extremely fine lithography is not needed anymore for the definition of the GPs, and the grafting of the brush layer is not constrained to the bottom of the trench.

After surface activation, a thick layer of a 1.5 wt. % solution in propylene glycol methyl ether acetate (PGMEA) of a random copolymer brush (PS_60%_-r-PMMA) was spin-coated at 1500 rpm for 30 s. The random BCP completely filled the gaps between the GPs, coating all surfaces. After a 5-min annealing step at 230 °C in a tube furnace with nitrogen atmosphere, the chips were rinsed again in PGMEA. This ensured that all the non-grafted brush was diluted and washed away, leaving behind a thin film of PS_60%_-r-PMMA attached to walls and bottom of the trenches. Subsequently, a 0.5 wt. % PGMEA solution of lamellar PS-b-PMMA (natural pitch *L*_0_ = 28 nm) was spin-coated at 1500 rpm for 30 s and annealed at 265 °C for 10 min in a tube furnace with a continuous flow of N_2_.

Atomic force microscopy (AFM) characterization showed that polymer thickness inside the trenches, including BCP and brush layer, filled the gap completely, regardless of the width of the gap of the GP (350 nm to 550 nm). In the areas outside the trenches, the BCP thin film was discontinuous, adopting the form of islands, due to the reduced thin film thickness obtained at 0.5 wt. %. Effective perpendicular alignment with respect to the GPs, demonstrates that the brush layer successfully covered bottom and trench walls (Figure 4b,c).

### 4.3. Selective PMMA Removal and Pattern Transfer

After DSA, PMMA domains were selectively removed by reactive-ion etching (RIE) in Ar/O_2_ plasma (AMS 110DE, Alcatel Micro Machining Systems, Annecy, France). ICP power was set at 200 W and chuck power at 5 W, eliminating PMMA from all gaps with enough selectivity to still leave ∼20 nm of PS unconsumed.

**Figure 6:**
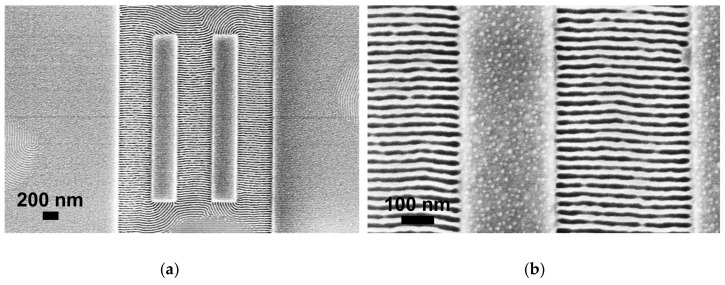
(**a**) SEM top-view micrograph of SiNWs obtained after pattern transfer of PS-b-PMMA; (**b**) zoomed-in micrograph.

Pattern transfer onto the device layer of the SOI substrate, using as mask the remaining PS (for nanowires) and SiO_2_ GPs (for membranes), was carried out in a mixed mode Bosch process based on SF_6_ and C_4_F_8_ for 18 s (Figure 6). ICP power was set at 1200 W and chuck power at 10 W.

**Figure 7:**
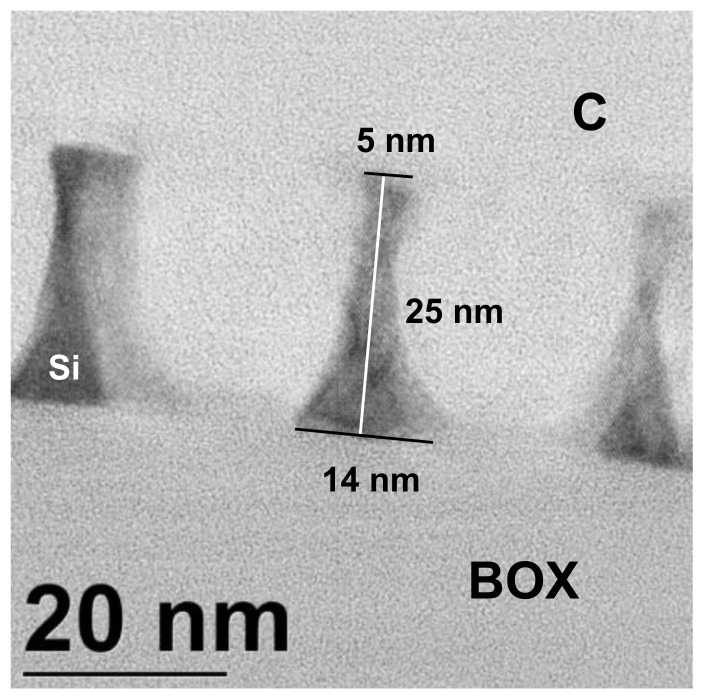
TEM micrograph of a lamella across several transferred SiNWs (before release). C serves as protective layer. SiNW height is determined by the thickness of the device layer of the SOI substrate.

As shown by transmission electron microscopy (TEM) characterization (Figure 7), nanowire dimensions are smaller than the expected half-pitch of the BCP, which indicates a certain degree of isotropy in the pattern transfer. This can be explained by a combination of several phenomena: inefficient passivation, poor diffusion, re-deposition of material and high electric field gradients that deflect ions towards the sidewalls [171,172,173].

### 4.4. Disposal of Undesired SiNWs

Microphase segregation after DSA not only took place in between GP sidewalls, where the BCP was guided in the proper direction, but also in areas with residual BCP thickness along the border of the GPs. To eliminate these undesired structures, additional EBL and RIE steps were performed.

To do so, PMMA 950K (2% in Anisole, MicroChem Corp., Westborough, MA, USA) was deposited at 2000 rpm for 1 min and baked for 1 min at 180 °C on a hot-plate. Rectangular areas where nanowires wanted to be removed were exposed by EBL at 20 kV, with an aperture of 20 μm and dose of 180 μC/cm^2^. Samples were developed for 30 s in a MIBK/isopropanol 1:3 solution and 30 s in isopropanol. After development, samples were dry etched for 20 s following the same recipe used for the pattern transfer onto silicon, and lastly, the PMMA mask was stripped in O_2_ plasma.

### 4.5. Release of the Structures

In the final stage, structures were released from the substrate in low-pressure gas-phase HF (SPTS µEtch), to avoid potential collapse. Prior to etching, samples were baked at 250 °C for 2 min to remove humidity. The process was performed by introducing 1250 sccm of N_2_, 300 sccm of EtOH and 310 sccm of HF at a pressure of 126 Torr. Processing time was ~3 min but independently adjusted for each chip, to guarantee full release of the structures in the design without completely releasing the GPs that serve as clamping.

**Figure 8:**
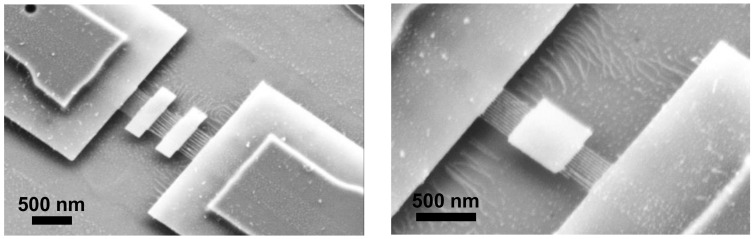
SEM micrographs of two of the multiple different silicon membranes obtained, suspended by high-density arrays of SiNWs.

Ultra-thin silicon membranes suspended by dense arrays of SiNWs were successfully achieved after the final release from the BOX (Figure 8). Likewise, the mask of HSQ from the GPs was also totally etched away.

Final obtained structures were composed of arrays of SiNWs with lengths between 350 nm and 500 nm, combined with either a single or a pair of silicon membranes. These were designed rectangular or squared-shaped, with lateral dimensions ranging from 0.5 µm to 5 µm. The systems, according to finite element analysis simulations, present Eigenfrequencies in the 25–200 MHz range, depending of individual dimensions. 

## 5. Conclusions

In this work we have introduced the opportunities that microphase separation of BCPs offers for patterning at the nanoscale. We have discussed how BCP microdomains placement and alignment can be directed by DSA methods, such as chemoepitaxy and graphoepitaxy, and demonstrated a process flow based on the latter for the fabrication of nanomechanical functional structures.

Obtained devices evidence DSA of BCPs enables the structuring of extremely high resolution features in a cost-effective manner. Moreover, the possibility of defining dense arrays of structures such as nanowires, which could be difficult by other high-resolution techniques like EBL due to proximity effects, is also proven.

DSA of BCPs is presently experiencing a delay in being adopted by high-volume manufacturing industries, like the semiconductor industry, due to difficulties in achieving a sufficiently low level of defectivity. However, we believe that DSA of BCPs is a viable solution to address application areas where the density of defects is not critical and the required functionality cannot be obtained by lithography with a reasonable cost of ownership. In this framework, we have addressed the manufacturing of nanoelectromechanical systems, and in particular dense arrays of suspended silicon nanowires, as an example of how DSA can be used in areas other than nanoelectronics. Although the process flow that we have developed combines DSA with electron beam lithography as a proof of concept (and therefore, with an overall low fabrication throughput), it can be easily adapted to other higher throughput top-down lithography techniques such as deep ultraviolet optical lithography. Other application areas will require similar efforts such as the ones presented here to take advantage of DSA integration into the manufacturing chain of functional nanometer scale devices.
